# Cotton Leaf Curl Multan Virus-Derived Viral Small RNAs Can Target Cotton Genes to Promote Viral Infection

**DOI:** 10.3389/fpls.2016.01162

**Published:** 2016-08-04

**Authors:** Jinyan Wang, Yafei Tang, Yuwen Yang, Na Ma, Xitie Ling, Jialiang Kan, Zifu He, Baolong Zhang

**Affiliations:** ^1^Jiangsu Key Laboratory for Bioresources of Saline Soils, Provincial Key Laboratory of Agrobiology, Jiangsu Academy of Agricultural SciencesNanjing, China; ^2^Guangdong Provincial Key Laboratory of High Technology for Plant Protection, Plant Protection Research Institute, Guangdong Academy of Agricultural SciencesGuangzhou, China

**Keywords:** viral small RNAs, cotton leaf curl virus, deep sequencing, vsiRNA targets, VIGS, *Geminiviridae*, begomovirus

## Abstract

RNA silencing is a conserved mechanism in plants that targets viruses. Viral small RNAs (vsiRNAs) can be generated from viral double-stranded RNA replicative intermediates within the infected host, or from host RNA-dependent RNA polymerases activity on viral templates. The abundance and profile of vsiRNAs in viral infections have been reported previously. However, the involvement of vsiRNAs during infection of the *Geminiviridae* family member cotton leaf curl virus (CLCuD), which causes significant economic losses in cotton growing regions, remains largely uncharacterized. Cotton leaf curl Multan virus (CLCuMuV) associated with a betasatellite called Cotton leaf curl Multan betasatellite (CLCuMuB) is a major constraint to cotton production in South Asia and is now established in Southern China. In this study, we obtained the profiles of vsiRNAs from CLCuMV and CLCuMB in infected upland cotton (*Gossypium hirsutum*) plants by deep sequencing. Our data showed that vsiRNA that were derived almost equally from sense and antisense CLCuD DNA strands accumulated preferentially as 21- and 22-nucleotide (nt) small RNA population and had a cytosine bias at the 5′-terminus. Polarity distribution revealed that vsiRNAs were almost continuously present along the CLCuD genome and hotspots of sense and antisense strands were mainly distributed in the Rep proteins region of CLCuMuV and in the C1 protein of CLCuMuB. In addition, hundreds of host transcripts targeted by vsiRNAs were predicted, many of which encode transcription factors associated with biotic and abiotic stresses. Quantitative real-time polymerase chain reaction analysis of selected potential vsiRNA targets showed that some targets were significantly down-regulated in CLCuD-infected cotton plants. We also verified the potential function of vsiRNA targets that may be involved in CLCuD infection by virus-induced gene silencing (VIGS) and 5′-rapid amplification of cDNA end (5′-RACE). Here, we provide the first report on vsiRNAs responses to CLCuD infection in cotton.

## Introduction

Cotton leaf curl disease (CLCuD) is a serious disease of cotton across Africa and South Asia (Rajagopalan et al., 2012). CLCuD is caused by a pathogen complex of monopartite begomoviruses (single-stranded DNA viruses of the family *Geminiviridae* that are transmitted by the whitefly *Bemisia tabaci*) and a specific DNA betasatellite (DNA-β) molecule, cotton leaf curl Multan betasatellite (CLCuMB; Briddon et al., 2001; Mansoor et al., 2003). The genomes of monopartite begomoviruses encode six genes, two in the sense orientation [encoding the coat protein (CP) and AV2 protein] and four in the complementary-sense orientation [encoding the replication-associated protein (Rep), the transcriptional-activator protein (C2), the replication enhancer protein (C3) and the C4 protein] (Rojas et al., 2001; Briddon et al., 2014). Betasatellites are a relatively new class of single strand DNA satellites that are required by their helper begomoviruses to symptomatically infect hosts species (Briddon et al., 2001; Briddon and Stanley, 2006). Betasatellites encode for a dominant symptom determinant (known as βC1) which is a suppressor of post-transcriptional gene silencing (PTGS). It may be involved in movement of the virus with in plant tissues and in increasing viral DNA levels in *planta* (Qazi et al., 2007; Amin et al., 2011).

RNA silencing is a highly conserved antiviral defense strategy in plant species that is known to control gene expression and subsequently regulate development, stress-induced responses and genome stability (MacLean et al., 2010). RNA silencing relies on short RNA (sRNA) molecules (21–24 nt). sRNA molecules are important mediators of RNA silencing associated pathways in nearly all eukaryotic organisms (Ruiz-Ferrer and Voinnet, 2009; Borges and Martienssen, 2015). There are two main classes of sRNAs in plants, microRNAs (miRNAs) and short interfering RNAs (siRNAs; Brosnan and Voinnet, 2009). These sRNAs are generated from either double-stranded RNA (dsRNA) or folded structures by dicer-like (DCL) proteins and guide Argonaute (AGO) proteins. These complexes are known as RNA-induced silencing complexes (RISC), which promote the silencing of mRNAs in a sequence-specific manner. Antiviral silencing can be triggered by viral dsRNA and highly structured single-stranded RNA (ssRNA), which can be recognized and cleaved by DCL proteins and processed into virus-derived small interfering RNAs (vsiRNA) in virus-infected plants (Hamilton and Baulcombe, 1999; Moissiard and Voinnet, 2006). These vsiRNAs can be considered indicative of RNAi machinery activation within the host to counteract viral infection. Virus-specific dsRNA, which is an RNAi trigger molecule, can be created by many different mechanisms in infected cells, including virus-encoded RNA polymerases, base pairing between plus and minus strands of viral RNAs, imperfect folding of self-complementary sequences and host-encoded RNA dependent RNA polymerase (RDR) action (Kasschau et al., 2007). Two different classes of vsiRNAs are produced during virus infections. These include primary siRNAs that are derived from DCL-mediated cleavage of initial trigger RNAs, and secondary siRNAs that require an RDR during their biogenesis (Shimura et al., 2011; Smith et al., 2011).

It was previously predicted that vsiRNAs could trigger antiviral defenses through PTGS, and exploit the host RNA silencing system to target complementary host transcripts (Zhang et al., 2015). Previous research suggests that viruses may use sRNAs to silence targeted host genes when there is close to perfect complementarity (Addo-Quaye et al., 2008). However, the potential role of viral siRNAs in regulating host gene expression has rarely been reported. Cucumber mosaic virus (CMV) Y satellite RNA (Y-sat) is a non-coding subviral RNA molecule that modifies typical symptoms induced by CMV in specific hosts; Y-sat causes a bright yellow mosaic on its natural host *Nicotiana tabacum*. Some siRNAs derived from Y-sat can target the mRNA of tobacco magnesium protoporphyrin chelatase subunit I (*chlI*, a key gene involved in chlorophyll synthesis) and downregulate its expression (Shimura et al., 2011; Smith et al., 2011). In another case, two peach latent mosaic viroid (PLMVd)-derived siRNAs specifically target host CHLOROPLASTIC HEAT-SHOCK PROTEIN 90 transcripts, leading to the albino phenotype of peach leaves infected with PLMVd (Navarro et al., 2012). vsiRNAs have also been reported to promote the silencing of host mRNAs in a sequence-specific manner using degradome analysis and 5′ RACE (Miozzi et al., 2013a).

Cotton leaf curl virus was first reported in the Guangxi and Guangdong provinces of China, which may be derived from proximity to other cotton growing areas of the world (Cai et al., 2010; Tang et al., 2015). Plants affected by CLCuD exhibit a series of abnormal symptoms including vein swelling, upward or downward cupping of leaves, and the formation of enations on the main veins of abaxial leaf surfaces (Sattar et al., 2013). Investigations of how viruses involved in CLCuD interact with the RNA silencing pathway have been limited compared to other Geminiviruses. However, some major advances have been made recently. For example, V2, C2, and C4 encoded by CLCuMuV and βC1 encoded by CLCuMuB exhibit suppressor activity (Qazi et al., 2007). This analysis also showed that CLCuMuV C4 and CLCuMuB βC1 can bind short RNAs, preferring the ds and ss forms, respectively. This suggests that these suppressors act to sequester siRNAs and subsequently prevent their incorporation into the RNA-induced silencing complex involved in sequence-specific mRNA degradation (Amin et al., 2011). However, the roles of vsiRNAs in the interaction between CLCuD and cotton are still unknown.

Deep sequencing or next generation sequencing (NGS) can provide insights into virus-induced plant defense mechanisms. Characterization of vsiRNAs by deep sequencing techniques has mostly been done in a few host plants, for, e.g., maize plants infected with Sugarcane Mosaic virus (Xia et al., 2014), tomato plants infected with Tomato yellow leaf curl virus (Yang et al., 2011), apple trees infected with *Apple stem grooving virus* (Visser et al., 2014), and rice plants infected with Rice stripe virus (Xu et al., 2012). In this study, the profile of vsiRNAs derived from CLCuD infected upland cotton (*Gossypium hirsutum*) plants was obtained by deep sequencing. The results point to significant differences in the vsiRNA abundance of CLCuD when infecting upland cotton plants. We also analyzed the characteristics of vsiRNAs and predicted the targets of vsiRNA by qRT-PCR and virus-induced gene silencing (VIGS). Our results indicate that a large number of vsiRNA play important roles in CLCuD infection.

## Materials and Methods

### Plant Growth and Virus Source

Upland cotton (*G. hirsutum*) line Zhongmian 35 plants were grown in growth chambers (28°C day and 22°C night, 16 h light and 8 h dark cycles) for plant growth and virus inoculation. CLCuMuV (accession number KP762786) was isolated from diseased cotton in the Guangdong province of China (Tang et al., 2015) and maintained at -80°C. Cotton plants at the two-leaf stage were inoculated with a CLCuD infectious clone pGreenII049-1.6A and pGreenII049-2.0β (Tang et al., 2015). At 20 days post-inoculation (dpi), the systemically infected leaves and non-inoculated leaves were harvested and pooled for DNA and RNA extraction. To ensure the success of CLCuD infection in the sequencing samples, SYBR PCR assay was performed on the qTOWER 2.0/2.2 (Analytik Jena, Germany) with the AceQ qPCR SYBR Green Master Mix (Vazyme, China) using the following PCR conditions: 5 min of denaturation at 95°C followed by 40 cycles of 95°C for 10 s, 60°C for 30 s. The primers of CP for detecting CLCuD virus content and cotton histone gene (AF024716) are listed in Supplementary Table S3 (Wan et al., 2016). Furthermore, at 60 dpi, newly developed leaves of selected cotton plants were used to observe the phenotype following CLCuD infection.

### Total Nucleic Acid Extraction and Small RNA Sequencing

DNA from infected and uninfected cotton leaves was extracted using the CTAB method as described (Wang et al., 2015). Total RNA was extracted with the Trizol reagent (Invitrogen, Carlsbad, CA, USA) for qRT-PCR as well as small RNA sequencing. For deep sequencing, the total concentration of RNA was quantified with the aid of a spectrophotometer (Nanodrop ND-2000, Thermo Fisher Scientific, USA), and RNA integrity was qualified using a Bio-Analyzer 2100 (Agilent Technologies, Germany). Total RNA was separated on 17% denaturing polyacrylamide gels and small RNAs of 15–36 nt were recovered. RNA adaptors were then ligated to the small RNAs followed by reverse transcription into cDNAs. The resulting cDNAs were amplified by PCR and subjected to Illumina Hiseq 2000 sequencing. Two independent biological replicates of infected and uninfected plants were used for RNA sequencing.

### Bioinformatic Analyses of Small RNA Sequences

Adapter sequences were removed from the raw data by a set of Perl scripts, and the reads were filtered for quality (quality score >20) using FASTX-toolkit (V0.0.13) ^1^. Small RNA sequences between 18 and 28 nt in length were extracted. Next, the filtered reads were mapped to the CLCuMuV (KP762786) and CLCuMuB (KP762787) genome, respectively, using Bowtie2 aligner software with two mismatches permitted (Langmead and Salzberg, 2012). The data discussed in this publication have been deposited in National Center for Biotechnology Information (NCBI’s) Sequence Read Archive^2^ with accession number SRS1423865.

### Real-Time Quantification of Viral RNA

For the siRNA expression assay, total RNA was extracted as described previously. cDNA for siRNA qPCR assays was prepared using the HiScript II 1st Strand cDNA Synthesis Kit (Vazyme) following a protocol implementing the looped RT-PCR method (Varkonyi-Gasic et al., 2007). Stem-loop primers of siRNAs (Supplementary Table S1) were designed for reverse transcription such that 6 bp at the 5′ end of the stem-loop primer were complementary to 6 bp at the 3′ end of the sRNA. qRT-PCR analysis was performed using a real-time PCR thermal cycler qTOWER 2.0/2.2 (Analytik Jena, Germany) with the following PCR conditions: denaturation for 10 s at 95°C, annealing for 15 s at 60°C, and extension for 20 s at 72°C for 35 cycles. The PCR efficiency and standard curve were analyzed to optimize the comparative threshold (2^-ΔΔCT^) method. U6 snRNA was used as an internal standard.

### Target Gene Prediction and qRT-PCR Analysis

In this study, we used the psRobot software and cotton EST sequences from NCBI to predict cotton mRNAs targeted by vsiRNA derived from CLCuD (Wu et al., 2012). CAP3 software was used to assemble the primitive EST sequences^3^. The following criteria were used as for this purpose: (1) No more than four mismatches between vsiRNA and target mRNA (G-U bases count as 0.5 mismatches); (2) No more than two adjacent mismatches in the vsiRNA/target duplex; (3) No adjacent mismatches in position 2–12 of the vsiRNA/target duplex (5′-terminus of vsiRNA); (4) No mismatches in position 10–11 of vsiRNA/target duplex; (5) No more than 2.5 mismatches in positions 1–12 of the vsiRNA/target duplex (5′-terminus of vsiRNA). The target EST sequences were predicted using BLASTX and checked against a non-redundant (NR) protein database and the Kyoto Encyclopedia of Genes and Genomes (KEGG) database with an *E*-value of 1e-10. Best hits were then used to validate target gene function and metabolic pathways that are regulated by vsiRNAs. The biological process and molecular function of target genes were identified using Interpro. Enrichment of gene ontology (GO) categories was conducted with an agriGO analysis toolkit^4^ using the TopGO ‘elim’ algorithm for the aspects ‘biological process’ and ‘molecular function.’ Selected categories were sorted from lowest to highest *P*-value (*P* < 0.01; Alexa et al., 2006; Du et al., 2010).

For expression pattern analysis of vsiRNA targets, RNA from young leaves was extracted with the PLANT simple RNA extraction kit (TIANGEN) and reverse transcribed by the HiScript II Q RT SuperMix for qPCR (Vazyme). Primers for qRT-PCR were designed using Primer5 software and primer specificity was evaluated by blasting primer sequences against the NCBI database (Supplementary Table S1). PCR amplifications were performed in a real-time thermal cycler qTOWER 2.0/2.2 (Analytik Jena, Germany) in 15 μl final reaction volumes containing 1.0 μl of cDNA, 0.5 μl each primer (10 μM), 6 μl of sterile water, and 7.5 μl (2×) SYBR Premix ExTaq^TM^ II Kit (TaKaRa, Japan). The conditions for amplification were as follows: 5 min of denaturation at 95°C followed by 40 cycles of 95°C for 10 s, 60°C for 20 s, and 72°C for 10 s. The histone gene of cotton (AF024716) was used as an internal standard (Wan et al., 2016). Relative gene expression was calculated using the 2^-ΔΔCT^ method (Livak and Schmittgen, 2001). All qRT-PCR expression assays were independently performed and analyzed using three biological replicates under identical conditions.

### VIGS Vector Construction and *Agrobacterium* Infiltration

Virus-induced gene silencing is a powerful tool for plant functional genomics, especially in plants that pose difficulties with genetic engineering. Our development of a cotton leaf crumple virus (CLCrV)-based VIGS vector provides an efficient gene-silencing tool that can be used for reverse genetics and genomics studies of candidate genes in cotton (Fu et al., 2015; Liu et al., 2015). To generate pCLCrV-vsiR3114-T, a 354 bp fragment of vsiR3114 target Contig28334 was amplified from upland cotton by PCR with the pair primers Contig28334 F SpeI and Contig28334 R PacI (Supplementary Table S1). The resulting PCR product was inserted into pCLCrVA to generate pCLCrV-vsiR3114-T. Empty vector-infiltrated plants were used as controls.

Transformed *Agrobacterium* cultures were grown until approximately OD_600_ = 1.0, and then incubated in induction buffer [10 mM MgCl_2_, 100 mM MES (pH 5.7), 100 M acetosyringone] for 3 h at room temperature. The combinations of the induced cultures were infiltrated into the abaxial side of cotyledon leaves of 2-week-old cotton seedlings. After *Agrobacterium* infiltration, cotton plants were kept in an incubator at 25°C with a relative humidity of 80%. Twenty days after *Agrobacterium* infiltration, cotton plants were injected with a CLCuD infectious clone. Ten days after *Agrobacterium* infiltration, new emerging leaves from the CLCuD infected plants were used to extract RNA and DNA, which were subsequently used to determine the expression levels of the Contig28334 and CLCuD DNA accumulation in the VIGS-treated plants by qRT-PCR, respectively. Conditions and parameters for qRT-PCR analyses were as described above.

### 5′-RACE Analysis

The vsiR3114 target Contig28334 mRNA cleavage sites was validated by modified 5′-RACE (Llave et al., 2002). Firstly, poly(A) mRNA was purified using Dynabeads^®^ mRNA purification Kit (Ambion). Then the fractionated poly(A) mRNA was ligated to the GeneRacer RNA Oligo adapter using the GeneRacer Kit (Invitrogen). The RNA was reversed transcribed using random decamers and then the 5′-end of cDNA was amplified using the GeneRacer 5′ outer primer and the Contig28334-specific reverse primer for the first PCR. The amplified product was used for subsequent nested PCR using the GeneRacer 5′ inner primer and the Contig28334-specific nested reverse primer. The amplified product was cloned into pEASY-Blunt (Transgen Biotech, China) for sequencing.

## Results

### vsiRNAs Characteristics

**FIGURE 1:**
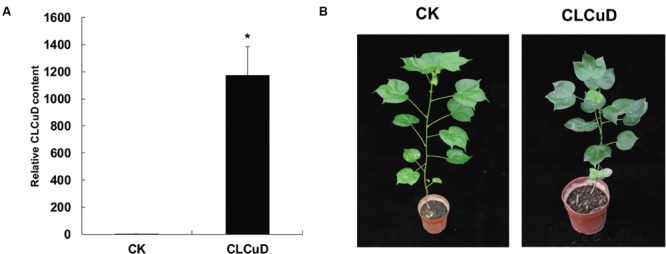
**Leaf phenotype and cotton leaf curl virus (CLCuD) virus accumulation after CLCuD infection of upland cotton plants. (A)** CLCuD accumulation in CK and CLCuD-inoculated cotton plants were estimated from total genomic DNA by qRT-PCR at 20 dpi. Values were normalized using the cotton histone gene (AF024716) as an internal reference. Error bars represent standard errors of three biological replicates and asterisk indicates the significant difference based on the Student’s *t*-test (*P* < 0.05). **(B)** Leaf phenotype after CLCuD infection at 60 dpi of the cotton plants.

**FIGURE 2:**
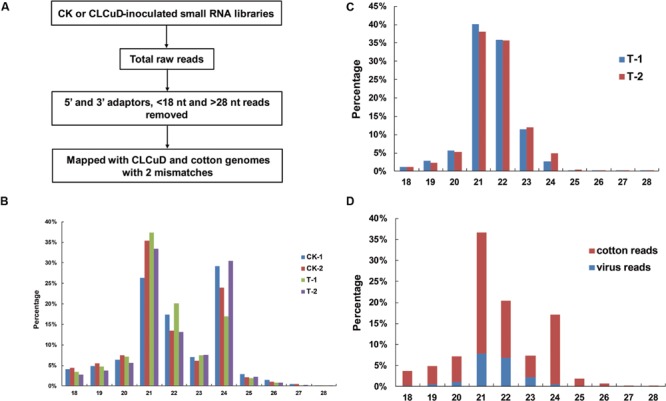
**Viral small RNAs (vsiRNAs) accumulate at high levels in CLCuD-inoculated cotton plants. (A)** A summary of the bioinformatics pipeline employed for the systematic identification of vsiRNAs reads from small RNA libraries recovered from CK and CLCuD-inoculated systemic leaves in cotton plants. **(B)** Size distribution of small RNAs in libraries prepared from CK and CLCuD-inoculated cotton plants with two biological replicates. CK-1 and CK-2 indicate two biological replicates of small RNA libraries without CLCuD-inoculated cotton plants. T-1 and T-2 indicate two biological replicates of small RNA libraries after CLCuD-inoculated cotton plants at 20 dpi. **(C)** Histogram representation of total vsiRNAs reads with two biological replicates in each size class. T-1 and T-2 indicate two biological replicates of small RNA libraries after CLCuD-inoculated cotton plants at 20 dpi. **(D)** Size distribution of total small RNAs in the library from CLCuD-inoculated cotton plants. Cotton reads indicate the reads mapped to the upland cotton genome. Virus reads indicate the reads mapped to the CLCuD genome.

The upland cotton line Zhongmian 35 was inoculated with a CLCuD infectious clone. At 20 dpi, the virus content of CLCuD was detected in newly formed leaves after CLCuD inoculation and it was found that virus accumulation increased in CLCuD-inoculated cotton leaves (**Figure 1A**), while the expression of reference histone gene is not affected before and after CLCuD infection. At 60 dpi, leaves were typically curly in comparison with wild type (**Figure 1B**). To obtain vsiRNAs profiles produced during CLCuD infection, sRNAs derived from cotton plants that were inoculated with CLCuD at 0 dpi (CK) and 20 dpi for two biological replicates were analyzed using deep sequencing on the Illumina Hiseq2000 platform. A total of 39,996,644 and 45,995,712 reads were gathered from the sRNA library of either CK or CLCuD-inoculated cotton plants, respectively (**Table 1**). Reads ranging from 18 to 28 nt were mapped to the viral genome in both sense and antisense orientations. Sequences within two mismatches were regards as vsiRNAs in the libraries (**Figure 2A**). In total, 5,082,943 vsiRNA reads were identified in CLCuD-inoculated cotton plants, accounting for 12.7% of 18–28 nt reads. However, only 5755 reads matched the CLCuD genome in the CK library, which corresponded to 0.01% of 18–28 nt reads (**Table 1**). In the CK and CLCuD-inoculated libraries, 21 and 24 nt small RNAs accumulated to high levels, representing 33 and 25% of total small RNAs, respectively (**Figure 2B**). In CLCuD-infected cotton plants, 21- and 22-nt vsiRNAs were most, representing 40 and 36% of total vsiRNAs, respectively (**Figure 2C**; Supplementary Table S2), which suggested that the cotton DCL4 and DCL2 proteins could be predominant dicer ribonucleases with roles in vsiRNA biogenesis. Interestingly, 21- and 22-nt small RNAs increase was primarily attributed to the accumulation of vsiRNAs (**Figure 2D**), suggesting that CLCuD inoculation generated high amounts of vsiRNAs, which was the result of antiviral RNA silencing or a specific CLCuD-host interaction.

**Table 1:** Summary of small RNA data.

	CK-1	CK-2	T-1	T-2
Total reads	17929502	22067142	21535598	24460114
Total number of mapped CLCU genome reads	2341	3414	3658518	1565280
Non-redundant number of reads mapped reads	2341	3414	3646356	1555670
The ratio of mapped CLCU genome reads (%)	0.01%	0.02%	16.99%	6.41%
Genome A coverage	none	none	85.24%	81.29%
Genome B coverage	none	none	76.60%	71.10%

*CK-1 and CK-2 were control samples from cotton plants before CLCuD infection; T-1 and T-2 were CLCuD-inoculated samples from cotton plants collected 20 days after CLCuD inoculation.*

### vsiRNAs Resulting from CLCuD Infection

**FIGURE 3:**
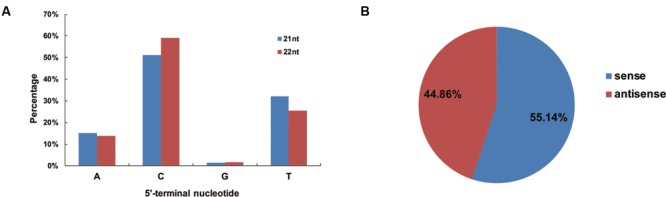
**Relative frequency of the 5′-terminal nucleotide of vsiRNAs and the accumulation of sense and antisense vsiRNAs. (A)** Relative frequency of distinct 5′-terminal nucleotides in 21- and 22-nt vsiRNAs of CLCuD-inoculated library. **(B)** Accumulation of sense and antisense vsiRNAs. Percentage for each class of vsiRNAs from the CLCuD-inoculated library is shown within the pie graph.

To determine potential interactions between vsiRNAs and different AGO complexes, the relative abundance of vsiRNAs was analyzed according to their 5′ terminal nucleotides, and it was found that the C nucleotide was the most abundant nucleotide (51 and 59%) at the 5′end for the 21- and 22-nt vsiRNAs (**Figure 3A**), respectively, while the U nucleotide was the least abundant. These results showed that 21- and 22-nt vsiRNAs may potentially load into diverse AGO-containing complexes with most of vsiRNAs preferentially loaded into AGO5 (Mi et al., 2008). To explore the origin of the vsiRNAs, the polarity distribution of vsiRNAs was further characterized. It was found that sense vsiRNAs (55.14%) levels were greater than antisense vsiRNAs (44.86%), suggesting that sense CLCuD RNA strands were predominant (**Figure 3B**).

**FIGURE 4:**
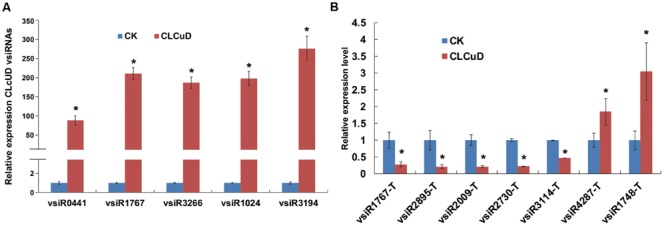
**The relative expression level of CLCuD vsiRNAs and predicted vsiRNA targets in CK and CLCuD-inoculated cotton plants. (A)** The relative expression level of CLCuD vsiRNAs by stem-loop reverse transcription PCR in CK and CLCuD-inoculated cotton plants. Values were normalized using the cotton U6 RNA as an internal reference. Error bars represent standard errors of three biological replicates and the asterisk indicates significant difference based on the Student’s *t*-test (*P* < 0.05). **(B)** The relative expression level of vsiRNA targets using qRT-PCR analysis in CK and CLCuD-inoculated cotton plants. The cotton histone gene (AF024716) was used as an internal reference. Error bars represent standard errors of three biological replicates, and asterisks indicate significant differences based on the Student’s *t*-test (*P* < 0.05).

There were large amounts of vsiRNAs accumulated in the host plants when virus infection triggered the RNA silencing mechanism. To confirm the existence of vsiRNAs, total RNA was used to analyze the selected five highly accumulated vsiRNAs derived from different CLCuD genome positions by stem-loop RT-PCR and quantitative RT-PCR (Salone and Rederstorff, 2015). As expected from the small RNA sequencing expression patterns, the qRT-PCR results were consistent with the deep sequencing results (**Figure 4A**). These results show that there are large amounts of vsiRNAs that accumulate in CLCuD-inoculated cotton plants when CLCuD virus infection triggered the RNA silencing mechanism.

### Hotspots for Generation of vsiRNA in the CLCuD Genome

**FIGURE 5:**
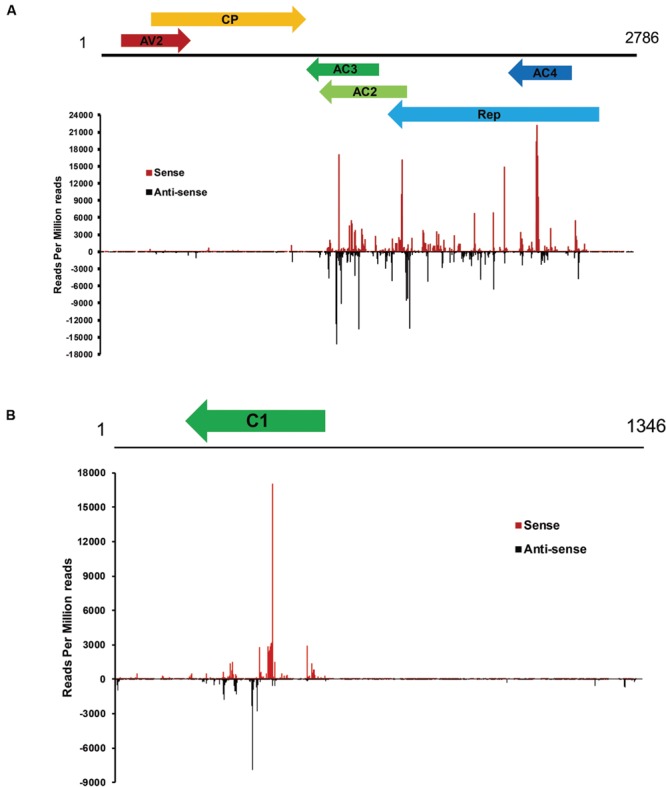
**Profile of vsiRNAs obtained from CLCuD-inoculated cotton plants. (A)** Schematic diagram of the CLCuMV genome and hotspots for vsiRNAs from CLCuD-inoculated cotton plants at single-nucleotide resolution. The graphs plot the number of vsiRNA reads at each nucleotide position of the 2786 CLCuMV genome. Red bars above the axis represent sense reads starting at each respective position and black bars below represent antisense reads ending at the respective position. **(B)** Schematic diagram of the CLCuMB genome and hotspots for vsiRNAs from CLCuD-inoculated cotton plants at single-nucleotide resolution. The graphs plot the number of vsiRNA reads at each nucleotide position of the 1346 CLCuMB genome. Red bars above the axis represent sense reads starting at each respective position and black bars below represent antisense reads ending at the respective position.

**FIGURE 6:**
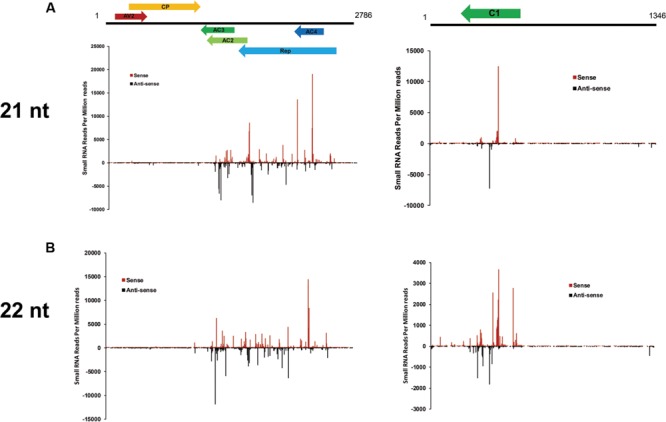
**Profile of 21- and 22-nt vsiRNAs obtained from CLCuD-inoculated cotton plants. (A)** Schematic diagram of CLCuD genome and hotspots for 21-nt vsiRNAs from CLCuD-inoculated cotton plants at single-nucleotide resolution. **(B)** Schematic diagram of the CLCuD genome and hotspots for 22-nt vsiRNAs from CLCuD-inoculated cotton plants at single-nucleotide resolution.

To examine the genomic distribution of vsiRNAs, all vsiRNAs of one CLCuD-inoculated sample derived from sense and antisense strands were mapped along the CLCuD genome. Sense and antisense vsiRNAs both displayed a clear non-uniform distribution pattern along the genome with a large proportion of vsiRNAs found in specific regions, such as AC3, AC2, AC4 and Rep proteins in CLCuMuV and C1 protein in CLCuMuB (**Figures 5A,B**). Further estimation of hotspots generated by vsiRNA in CLCuMuV showed that this region corresponded to Rep in CLCuMuV (covering about 40% of CLCuMuV genome), and had a tendency to contain higher levels of vsiRNAs with more than 70% of sequenced vsiRNAs clustered in this region. In contrast, the intergenic region had a tendency to accumulate lower levels of vsiRNAs with only about 0.1% of vsiRNAs located in this region both in the CLCuMuV and CLCuMuB genomes (**Figures 5A,B**), indicating that the CLCuD genome contained regions that serve as preferential targets of vsiRNA production. This trend was consistent with another CLCuD-inoculated small RNA library (Supplementary Figure S1). Furthermore, the most prominent peak of sequence abundance corresponding to 21-nt vsiRNAs typically localized to the same genomic regions as identification of the peak corresponding to 22-nt vsiRNAs, indicating that DCLs have a similar targeting preference toward regions of the CLCuD genome (**Figure 6**).

### Plant Transcripts Targeted by vsiRNAs

**FIGURE 7:**
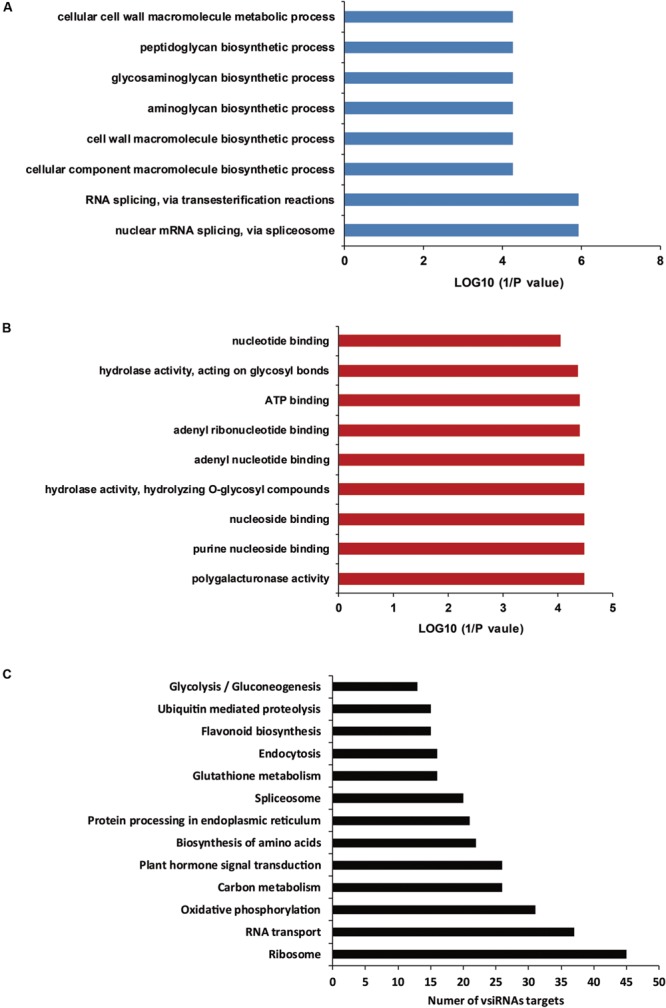
**Gene ontology (GO) enrichment analysis and KEGG pathways of vsiRNA targets. (A)** GO enrichment analysis of vsiRNA targets involved in various biological processes. **(B)** GO enrichment analysis of vsiRNA targets involved in various molecular functions. **(C)** The vsiRNA targets involved in KEGG pathways.

In a previous report, vsiRNA were shown to target host genes with weak base pairings as a match pattern (Smith et al., 2011). PsRobot is a web-based tool that is easy to use and is dedicated to the identification of the target for miRNAs (Wu et al., 2012). In this study, we used this software to identify cotton mRNAs targeted by vsiRNAs derived from CLCuD. All cotton EST sequences downloaded from NCBI were assembled using the CAP3 software, and were consequently used as targets for prediction of vsiRNAs. The vsiRNAs with RPM >10 and shared with two CLCuD-inoculated biological replicates were used to predict targets. Potential vsiRNAs displayed perfect or near-perfect complementarity to their predicted target genes. A total of 2462 potential targets were identified for 1723 vsiRNAs by searching for assembled cotton EST sequences (Supplementary Table S4). For most vsiRNAs, more than one potential target gene was predicted. To understand the roles of the predicted vsiRNA target genes in cotton, their sequences were used to query the NR, KEGG, and GO databases. A total of 2437 EST sequences were annotated via alignment with the NR protein database. The detailed annotation results of best BLASTX hits are provided in Supplementary Table S4. From the annotation, we found that a total of 28 predicted miRNA targets in cotton are transcription factors, including auxin-induced proteins, bHLH, WRKY, BZIP, MYB, F-box family, and Zinc finger proteins, many of which play important roles in plant growth and development. In addition to transcription factors, GO enrichment analysis revealed that predicted target genes were involved in a broad range of biological processes and molecular functions, and the most affected functions were associated with biosynthetic and metabolic processes (**Figures 7A,B**). A total of 276 metabolism networks were identified following KEGG enrichment of pathways with predicted vsiRNA target genes (Supplementary Table S5). These pathways include ribosome, RNA transport, oxidative phosphorylation, carbon metabolism, and plant hormone signal transduction (**Figure 7C**). This wide target range suggested that the identified vsiRNAs played significant roles in CLCuD-inoculated cotton plants.

It is established that siRNAs down-regulate targets at the post-transcriptional level. To determine whether vsiRNAs from CLCuD promoted the degradation of target transcripts in cotton, qRT-PCR was performed to investigate target transcript accumulation. Due to the vast amounts of vsiRNAs, only a few vsiRNAs with highly abundant reads were selected and their targets were subsequently validated by qRT-PCR. The results showed that targets of vsiR1767, vsiR2895, vsiR2009, vsiR3114, and vsiR2730 were significantly down-regulated in CLCuD-infected cotton plants, whereas the targets of vsiR4287 and vsiR1748 were up-regulated (**Figure 4B**). These results indicate that some vsiRNA targets may be involved in several pathways rather than only be regulated by vsiRNAs at the post-transcriptional level.

**FIGURE 8:**
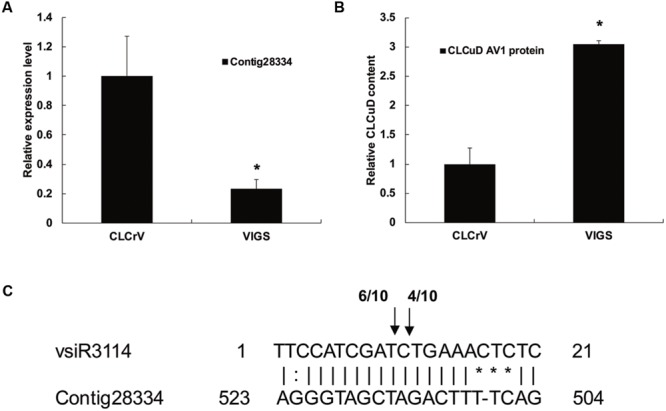
**The role of the vsiRNA target candidate during CLCuD infection with virus-induced gene silencing. (A)** Relative expression levels of vsiR3114 target Contig28334 using qRT-PCR analysis in the VIGS-treated cotton plants 20 days after *Agrobacterium* infiltration with CLCrV vectors. The cotton histone gene (AF024716) was used as an internal reference. Error bars represented standard errors of threeto clari biological replicates, and asterisks indicate significant differences based on the Student’s *t*-test (*P* < 0.05). **(B)** CLCuD accumulation in the Contig28334 silenced plants was estimated from the total genomic DNA by qRT-PCR. Values were normalized using the cotton histone gene (AF024716) as an internal reference. Error bars represented standard errors of three biological replicates and asterisk indicates significant difference based on the Student’s *t*-test (*P* < 0.05). **(C)** Validation of vsiR3114-guided target cleavage by 5′-RACE analysis. The number of 5′-RACE clones corresponding to each site is indicated by arrows.

To determine vsiRNA target function in CLCuD infection of cotton, we attempted to silence the expression of vsiR3114 target Contig28334 which encoded the MYB transcription factors. First, we cloned a 354 bp fragment of Contig28334 into pCLCrVA to produce pCLCrV-Contig28334. Then the CLCrV vector carrying fragments of Contig28334 were injected into plants by *Agrobacterium* infiltration at the cotyledon stage. Twenty days after *Agrobacterium* infiltration, cotton plants were injected with a CLCuD infectious clone. qRT-PCR assays revealed that the expression level of Contig28334 after silencing decreased by over 30% compared to the negative control (**Figure 8A**). Total genomic DNA of CLCuD-infected cotton plants was extracted for the detection of CLCuD virus accumulation after VIGS. The level of CLCuD accumulation in the VIGS-treated cotton plants was three times as much as that in the negative control plants, in which VIGS was not induced (**Figure 8B**).

To clarify whether the vsiR3114 target Contig28334 is cleaved by the vsiR3114, we analyzed the 5′ ends of cleaved mRNA products using 5′-RACE analysis. Sequencing of 5′-RACE products revealed two different cleavage sites in the Contig28334 mRNA (**Figure 8C**). These findings indicate that vsiRNA targets are involved in the response to CLCuD infection and may perform previously unknown functions in the CLCuD regulatory network.

## Discussion

RNA silencing is a small RNA-mediated repression mechanism that underlies gene regulation in a variety of eukaryotic organisms and plays a critical role in defense against viruses in host plants. Virus infection triggers plant PTGS resulting in the production of vsiRNAs in infected plant cells. NGS and computational methods have provided the technology platforms to identify an increasing number of small non-coding RNAs. In this study, a deep sequencing approach was employed to profile vsiRNAs from CLCuD-inoculated upland cotton plants. We also describe the first high-resolution sRNA map from CLCuMuV and CLCuMuB infected plants.

Sequence analysis of small RNA data demonstrated that CLCuD infection triggered the generation of large amounts of vsiRNAs, which accounted for 12.7% of 18–28 nt reads. Previous studies showed that DCL4-dependent 21-nt vsiRNAs are the predominant anti-viral silencing component in other Geminiviruses (Bouché et al., 2006; Garcia-Ruiz et al., 2010). In tomato yellow leaf curl Sardinia virus infected tomato plants, vsiRNAs of 21 nt (56%) and 22 nt (31%) predominated on a length basis (Miozzi et al., 2013b). Following *cabbage leaf curl virus* (CaLCuV) infection, 21 and 24 nt vsiRNAs represented the top 2 largest fractions of 20–25 nt viral reads, respectively (Aregger et al., 2012). However, this pattern differs from tomato yellow leaf curl China virus (TYLCNV) infections, in which 22 nt vsiRNAs accumulated preferentially in infected *Solanum lycopersicum* and *N. benthamiana* plants. Different size class distribution of vsiRNAs suggested that there is a difference between biosynthetic pathways of siRNAs in different virus inoculated plants. In the current study we observed after CLCuD infection that the population of 21-nt vsiRNAs is slightly higher than the 22-nt counterpart. These results suggest that DCL4 and DCL2 work synergistically in the production of vsiRNAs and there is a shift in the size of small RNAs after CLCuD inoculation.

Previously, 5′-terminal sRNA nucleotides were found to direct their specific sorting into distinct AGO complexes (Mi et al., 2008; Takeda et al., 2008; Wu et al., 2009). The majority of 21 and 22 nt vsiRNAs in our sequencing data showed that the 5′ nucleotide on 21 nt CLCuD vsiRNAs was most frequently a cytosine(C; 51%) followed by uracil (U; 32%) and adenine (A; 15%) (**Figure 3**). The analysis of the tomato yellow leaf curl Sardinia virus infected tomato small RNA data has indicated a preference for C as vsRNA 5′-terminal nucleotides, especially in antisense strand (Miozzi et al., 2013b). The low proportion of vsiRNAs beginning with a G nucleotide in our data sets is consistent with the absence of AGO proteins known to prefer siRNAs containing a 5′ terminal G (Mi et al., 2008). Furthermore, AGO1 played a dominant role in defending against plant viruses (Zhang et al., 2006; Qu et al., 2008). In CLCuD-infected cotton plants, vsiRNAs with a 5′ terminal U, which would be loaded into AGO1, accounted for the second most frequently observed proportion, suggesting that this might be an important mechanism of RNA silencing against CLCuD.

Polarity distribution analysis of sequenced vsiRNA accu mulation demonstrated that there is an approximately equal ratios of sense and antisense vsiRNAs (**Figure 5**), indicating that vsiRNAs could be produced from dsRNA precursors comprised of sense and antisense strands derived from the CLCuD genome. Also, hotspots for sense and antisense strands were clustered in gene-containing regions of the CLCuD genome (**Figure 5**). In a previous study, a slightly higher GC content in hotspots of the virus was investigated compared to other viral genomic regions (Donaire et al., 2009). However, in our study, no easily explainable relationship was observed between GC distribution and vsiRNAs hotspots (data not shown). We also found that there are some vsiRNAs derived from non-hotspot regions for both CLCuMuV and CLCuMuB. These vsiRNAs may be generated and then degraded by yet unknown mechanisms, which warrants further investigation.

Previous studies have suggested that vsiRNAs can silence specific host mRNAs when there is a near perfect comple mentarity at the post-transcriptional level (Shimura et al., 2011; Smith et al., 2011). However, a detailed investigation of interactions between CLCuD vsiRNAs and host targets is lacking. In this study, the psRobot software predicted some vsiRNA targets by expression analysis of vsiRNA targets, indicating that most of the predicted vsiRNA targets were down-regulated. This suggests that viral-induced gene silencing was the main regulatory mechanism in CLCuD-infected cotton plants. However, some vsiRNA targets were not down-regulated after CLCuD infection, which implies that there may be additional regulatory pathways besides cleavage of mRNAs, such as by translation inhibition (Brodersen et al., 2008; Li et al., 2013). Moreover, CLCuD infection may induce over-expression of some vsiRNA targets in non-RNA silencing pathways (Miozzi et al., 2013a). In our study, we found that vsiR3114 can target a cotton gene Contig28334 and the expression patterns between vsiR3114 and its target Contig28334 showed a negative correlation. After CLCuD infection, the vsiR3114 may lead to decreased expression of the Contig28334. In addition, the target of vsiR3114, Contig28334 was predicted to be an MYB transcription factor. In plants, MYB transcription factors have a highly conserved DNA-binding domain, which consists of up to four imperfect amino acid sequence repeats with 52 amino acids. Some MYB transcription factors have been reported to function in regulatory networks in defense against pathogens. MYB30 in *Arabidopsis* encodes an activator of the hypersensitive cell death program in response to pathogen attack (Raffaele et al., 2008). In addition, AtMYB102 and AtMYB72 play important roles in resistance against insects (De Vos et al., 2006) and beneficial fungi and bacteria (Segarra et al., 2009), respectively. In upland cotton, a defense-related MYB108 participates in the defense response against *Verticillium dahliae* infection (Cheng et al., 2016). Here, we provide evidence that the vsiRNA derived from CLCuMV can target host genes in response to CLCUD infection by VIGS and 5′-RACE analysis. However, more studies need to be conducted to determine the regulation pattern of vsiRNAs and their targets.

Even though our results indicate that cotton vsiRNAs play a key role in cotton antiviral RNA silencing, the molecular mechanisms underlying this phenomenon are unknown. Further research on the biological roles and functional mechanisms underlying specific CLCuD vsiRNAs need to be conducted in the future to systematically extend our understanding of CLCuD vsiRNAs functions.

## Author Contributions

JW, YT, ZH, and BZ performed the data analysis and drafted the manuscript. JW, YY, and XL participated in the analysis of the data. JW, YT, and XL performed the experiments. All authors have read and approved the final version of the manuscript.

## Conflict of Interest Statement

The authors declare that the research was conducted in the absence of any commercial or financial relationships that could be construed as a potential conflict of interest.

## Footnotes

1 http://hannonlab.cshl.edu/fastx_toolkit/

2 http://www.ncbi.nlm.nih.gov/sra

3 http://seq.cs.iastate.edu/cap3.html

4 http://bioinfo.cau.edu.cn/agriGO/
