# Supplementary material for: Cotton Leaf Curl Multan Virus-Derived Viral Small RNAs Can Target Cotton Genes to Promote Viral Infection
Source: Front Plant Sci. 2016 Aug 4;7:1162. doi: 10.3389/fpls.2016.01162 (PMC4972823; doi:10.3389/fpls.2016.01162)
Supplement: Supplementary file 2 [file Image_1.PDF]

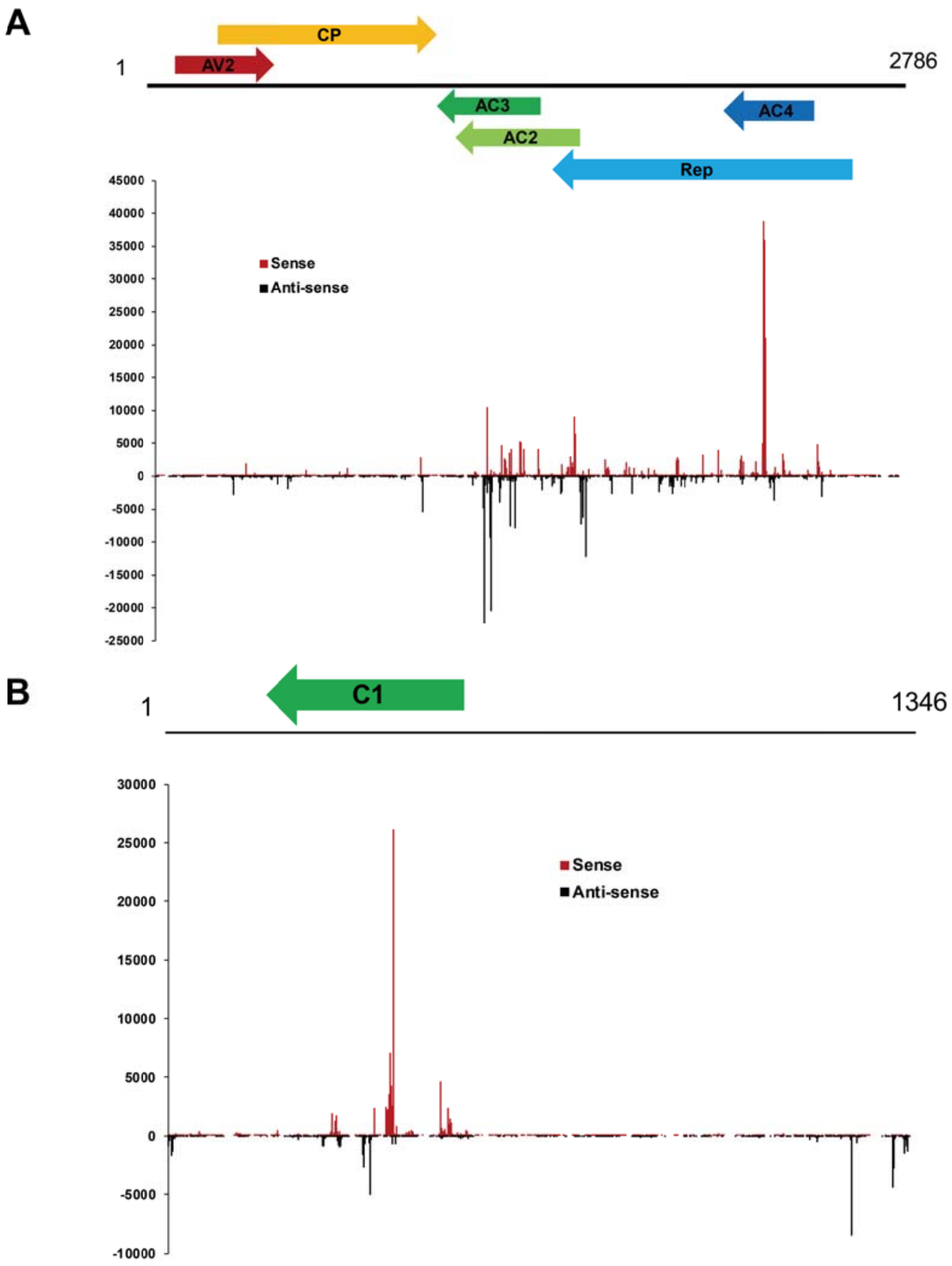

Supplementary Figure 1. Profile of vsiRNAs obtained from CLCuD-inoculated cotton plants with another biological replicate. A. Schemiatic diagram of the CLCuMV genome and hotspots for vsiRNAs from CLCuD-inoculated cotton plants at single-nucleotide resolution. The graphs plot the number of vsiRNA reads at each nucleotide position of the 2786 CLCuMV genome. Red bars above the axis represent

sense reads starting at each respective position and black bars below represent antisense reads ending at the respective position. B. Schemiatic diagram of the CLCuMB genome and hotspots for vsiRNAs from CLCuD-inoculated cotton plants at single-nucleotide resolution. The graphs plot the number of vsiRNA reads at each nucleotide position of the 1346 CLCuMB genome. Red bars above the axis represent sense reads starting at each respective position and black bars below represent antisense reads ending at the respective position.
